# Treatment outcomes and relapse in patients with *Mycobacterium avium-intracellulare* complex pulmonary disease

**DOI:** 10.1128/spectrum.01640-23

**Published:** 2023-09-27

**Authors:** Chia-Ling Chang, Chong-Jen Yu, Po-Ren Hsueh, Jung-Yien Chien

**Affiliations:** 1 Department of Internal Medicine, National Taiwan University Hospital Hsin-Chu branch, National Taiwan University College of Medicine, Hsinchu, Taiwan; 2 Graduate Institute of Clinical Medicine, National Taiwan University College of Medicine, Taipei, Taiwan; 3 Department of Internal Medicine, National Taiwan University Hospital, National Taiwan University College of Medicine, Taipei, Taiwan; 4 Department of Laboratory Medicine, National Taiwan University Hospital, National Taiwan University College of Medicine, Taipei, Taiwan; 5 Department of Laboratory Medicine, China Medical University Hospital, Taichung, Taiwan; 6 Department of Internal Medicine, China Medical University Hospital, Taichung, Taiwan; 7 School of Medicine, China Medical University, Taichung, Taiwan; 8 Ph.D Programme for Aging, College of Medicine, China Medical University, Taichung, Taiwan; Johns Hopkins University School of Medicine, Baltimore, Maryland, USA

**Keywords:** *Mycobacterium avium-intracellulare *complex pulmonary disease, treatment, sputum smear conversion, microbiological cure, relapse

## Abstract

The treatment responses and outcomes in patients with *Mycobacterium avium-intracellulare* complex pulmonary disease (MAC-PD) remain uncertain. This retrospective study was conducted in Taiwan between 2011 and 2020. A microbiological cure was defined as the requirement for a minimum of three consecutive negative cultures following culture conversion that continued until the completion of anti-MAC treatment. A total of 97 patients with MAC-PD were enrolled in this study. The sputum smear-negative conversion rate was 40% (19/47). Thirty (31%) patients achieved sputum culture-negative conversion rates within 3 years after treatment initiation. All patients with negative sputum culture conversion rates achieved microbiological cure. Patients treated with a macrolide + rifamycin + ethambutol (M + R + EMB)-based regimen had a higher microbiological cure rate than the other patients (39% vs 17%, *P* = 0.023). Patients with persistently positive sputum smears after 6 months of treatment had a lower microbiological cure rate than those with negative sputum smears (6% vs 44%, *P* < 0.001). Among 30 patients with microbiological cure, the median time from sputum culture conversion to treatment completion was 221.5 (0–483) days, and the 1-year relapse rate was 17%. Treatment with the M + R + EMB-based regimen was associated with a higher microbiological cure rate, and patients with persistently positive sputum smears after 6 months of treatment had a lower microbiological cure rate.

**IMPORTANCE:**

The treatment responses and outcomes in patients with *Mycobacterium avium-intracellulare* complex pulmonary disease (MAC-PD) remain uncertain. In this study, patients with MAC-PD treated with a macrolide + rifamycin + ethambutol (M + R + EMB)-based regimen had a higher microbiological cure rate than those treated with other regimens. After 6 months of treatment, patients with persistently positive sputum smears had a lower microbiological cure rate than those with negative sputum smears. Among patients with microbiological cure, the median time from sputum culture conversion to treatment completion was 221.5 days (range, 0–483), and the 1-year relapse rate was 17%.

## INTRODUCTION

The incidence and prevalence of nontuberculous mycobacterial pulmonary disease (NTM-PD) are increasing (1), and *Mycobacterium avium complex* (MAC) is the most common pathogen in Taiwan and worldwide (2, 3). Although the mortality rate of MAC-PD is high (4), determining the appropriate timing of treatment initiation in patients with MAC-PD remains challenging. Patients with MAC in the lungs do not necessarily require anti-MAC treatment. The potential risks and benefits of treatment initiation depend on the severity of the disease, risk of disease progression, and presence of comorbidity (5). Risk factors associated with disease progression include host [low body mass index (BMI) and presence of comorbidities], radiographic (fibrocavitary disease and extent of disease), laboratory (elevated inflammatory markers and hypoalbuminemia), and microbial (bacterial load and species) factors (6, 7).

For antimicrobial therapy, the ATS/ERS/ESCMID/IDSA Clinical Practice Guidelines recommend using at least three drugs, including a macrolide, for at least 12 months after culture conversion (8). Treatment regimens without macrolides are associated with lower sputum culture-negative conversion and higher mortality (9). However, this recommendation for treatment regimen and duration is not based on evidence from randomized controlled trials, and adherence to prescription of the recommended regimen is inconsistent. Studies of treatment outcomes of MAC-PD have found a 40%–80% sputum culture conversion rate (10) and 68.1% treatment success rate (11). Pharmacokinetic analyses have shown that the peak serum concentrations of the currently recommended key drugs, such as clarithromycin, azithromycin, and ethambutol, are frequently below the target range and that co-administration of rifampin decreases peak serum concentrations of macrolides (12). This might explain why treatment according to the guidelines yields limited cure rates. Adverse effects, expensive treatment, and prolonged treatment duration are common problems (13). Relapse of MAC-PD after successful treatment is common (14). New anti-MAC regimens, treatment durations, and drugs should be investigated further to improve the treatment of MAC-PD.

The long-term outcomes and relapse following different MAC-PD combination therapies are poorly understood. The aim of this study was to investigate the treatment outcomes, effectiveness of different treatment regimens, and disease relapse rates in patients with MAC-PD.

## RESULTS

A total of 97 patients treated for MAC-PD were enrolled, and 44% (43/97) were men. The median age was 61.1 years (range, 24.4–87.4 years), and the median BMI was 18.6 kg/m^2^ (range, 10.7–26.9 kg/m^2^). One patient with a BMI of 10.7 kg/m^2^ had a height of 153 cm and a weight of 25 kg. The main underlying diseases were hypertension (17%), old pulmonary tuberculosis (16%), and chronic obstructive pulmonary disease (10%). The most common radiographic pattern (70%, 68/97) was nodular bronchiectasis. The 3-year all-cause mortality rate was 7% (7/97) (Table 1). Among the seven patients who died within 3 years, two died from MAC-PD, one from lung cancer, one from buccal cancer, one from chronic obstructive pulmonary disease, one from bronchiectasis, and one from heart failure.

**TABLE 1:** Clinical characteristics of patients with *Mycobacterium avium-intracellulare* complex pulmonary disease^*a*^

	All (*n* = 97)	Non-microbiological cure (*n* = 67)	Microbiological cure (*n* = 30)	*P* value
Male	44.3 (43/97)	44.8 (30/67)	43.3 (13/30)	1
Age (years)	61.1 (24.4–87.4)	60.6 (24.4–87.4)	62.9 (25.8–80.6)	0.888
BMI^*b*^	18.6 (10.7–26.9) *n* = 89	19.1 (11.8–26.9) *n* = 63	18.0 (10.7–25.9) *n* = 26	0.457
Ex- or current smoker	15.9 (7/44)	12.9 (4/31)	23.1 (3/13)	0.404
Asthma	6.2 (6/97)	9.0(6/67)	0 (0/30)	0.173
COPD	10.3 (10/97)	11.9 (8/67)	6.7 (2/30)	0.719
Old pulmonary tuberculosis	15.5 (15/97)	16.4 (11/67)	13.3 (4/30)	0.772
Lung cancer	3.1 (3/97)	4.5 (3/67)	0 (0/30)	0.55
Hypertension	16.5 (16/97)	17.9 (12/67)	13.3 (4/30)	0.769
Coronary artery disease	4.1 (4/97)	1.5 (1/67)	10.0 (3/10)	0.086
Transplantation	2.1 (2/97)	1.5 (1/67)	3.3 (1/30)	0.525
Immunosuppressant	1.0 (1/97)	1.5 (1/67)	0 (0/30)	1
HIV infection	7.2 (7/97)	6.9 (6/67)	3.3 (1/30)	0.431
Type 2 diabetes mellitus	2.1 (2/97)	3.0 (2/67)	0 (0/30)	1
GERD	4.1 (4/97)	4.5 (3/67)	3.3 (1/30)	1
Rheumatic arthritis	2.1 (2/97)	3.0 (2/67)	0 (0/30)	1
Image				0.869
Fibro-cavity	12.4 (12/97)	13.4 (9/67)	10.0 (3/30)	
Nodular bronchiectasis	70.1 (68/97)	68.7 (46/67)	73.3 (22/30)	
Mixed type	17.5 (17/97)	17.9 (12/67)	16.7 (5/30)	
Subspecies				0.624
MAsH	25.8 (25/97)	26.9 (18/67)	23.3 (7/30)	
MIsI/C	47.4 (46/97)	49.3 (33/67)	43.3 (13/30)	
Others	26.8 (26/97)	23.9 (16/97)	33.3 (10/30)	
M + R + EMB-based regimen	62.9 (61/97)	55.2 (37/67)	80.0 (24/30)	0.024
Time from diagnosis to treatment (days)	79.0 (0–1866)	56 (0–1715)	105 (4–1866)	0.288
Time from treatment to sputum culture conversion (days)	125.5 (26–422)		125.5 (26–422)	
Duration >1 year	6.7 (2/30)		6.7 (2/30)	
Treatment time (days)	318 (30–910)	256 (30–910)	399 (169–636)	0.018
Duration >1 year	42.3 (41/97)	34.3 (23/67)	60.0 (18/30)	0.026
Time from sputum culture conversion to complete treatment (days)	221.5 (0–483)		221.5 (0–483)	
3-year image progression	11.6 (11/95)	16.9 (11/65)	0 (0/30)	0.015
3-year all-cause mortality	7.2 (7/97)	6.0 (4/67)	10.0 (3/30)	0.673

^*a*^ Data are presented as percentages (numerator/denominator) or medians (minimal–maximal).

^*b*^ BMI, body mass index; COPD, chronic obstructive pulmonary disease; GERD, gastroesophageal reflux disease; HIV, human immunodeficiency virus; MAsH, *Mycobacterium avium* subspecies *hominissuis*; MIsI/C, *Mycobacterium intracellulare* subspecies *intracellulare/chimaera*.

Of the 97 antibiotic-naïve patients with MAC-PD, 61 (63%) were treated with a macrolide + rifamycin + ethambutol (M + R + EMB)-based regimen (azithromycin/clarithromycin, rifampin/rifabutin, and ethambutol). The prescribed medications are shown in Table S1. The median time from diagnosis to treatment was 79 days (range, 0–1,866 days). The median treatment duration was 318 days (range, 30–910 days). The sputum smear conversion rate was 40% (19/47 patients), and the median time from treatment to negative conversion in the sputum smear was 111 days (range, 7–438 days). The culture-negative conversion rate was 31% (30/97 patients), and the median time from treatment initiation to sputum culture conversion was 125.5 days (range, 26–422 days). The median time from negative culture conversion to treatment completion was 221.5 days (range, 0–483 days) (Table S2). The 3-year microbiological cure rate was 31%, and 42% of patients received treatment for more than 1 year. Those who achieved cure had a significantly longer median treatment duration (399 days vs 256 days, *P* = 0.018). No significant differences were observed in the imaging patterns, including fibrocavity, nodular bronchiectasis, and mixed type, between the microbiological cure and non-microbiological cure groups (*P* = 0.869). The 3-year image progression rate evaluated using radiographic scores was higher in the non-microbiological cure group (*P* = 0.015) (Table 1).

Most patients (94%, 91/97 patients) were treated with macrolides (azithromycin or clarithromycin) (Table 2). The medications prescribed for the six patients who did not receive a macrolide-containing regimen are shown in Table S3. The microbiological cure rate was not significantly different between clarithromycin- and azithromycin-containing regimens (*P* = 0.655). Among 50 patients with MAC-PD who received clarithromycin, 38% (19/50) were prescribed a daily dose of 500 mg, and 62% (31/50) received a daily dose of 1,000 mg. There was no significant difference in median body weight between the clarithromycin 500 mg/day and clarithromycin 1,000 mg/day groups (52.0 kg vs 47.5 kg, *P* = 0.263). The median time from treatment initiation until sputum culture conversion was 98.5 days (range, 26–422 days), with median times of 388 days and 79 days in the 500 mg/day and 1,000 mg/day clarithromycin groups, respectively (*P* = 0.549). Kaplan-Meier analysis showed that patients with MAC-PD treated with clarithromycin 1,000 mg/day had similar rates of culture-negative conversion and microbiological cure to those treated with clarithromycin 500 mg/day (log-rank test *P* = 0.095 and 0.103, respectively). (Fig. S1A and B). Two patients experienced relapse. One patient received a clarithromycin dose of 1,000 mg/day, and the other received 500 mg/day. Among 41 patients with MAC-PD treated with azithromycin, the daily dose of azithromycin was 250 mg/day in 17% (7/41) patients and 500 mg/day in 83% (34/41) patients. The group receiving an azithromycin dose of 500 mg/day had a higher median body weight than the group receiving a dose of 250 mg/day (49.5 kg vs 39.5 kg, *P* = 0.015). The median time from treatment initiation to sputum culture conversion was 155 days (range: 61–300 days), with median times of 155 and 216 days (*P* = 0.465) in the azithromycin 500 mg/day and azithromycin 250 mg/day groups, respectively. Kaplan-Meier analysis showed no significant difference in culture-negative culture conversion and microbiological cure rates between patients receiving azithromycin 500 mg/day and those receiving 250 mg/day (log-rank test, *P* = 0.578 and 0.669, respectively) (see Fig. S1C and D). All three patients who experienced relapse received an azithromycin dose of 500 mg/day.

**TABLE 2:** Treatment drugs and treatment outcomes in patients with *Mycobacterium avium-intracellulare* complex pulmonary disease^*a*^

Drug	All (*n* = 97)	Non-microbiological cure (*n* = 67)	Microbiological cure (*n* = 30)	*P* value
M	93.8 (91/97)	92.5 (62/67)	96.7 (29/30)	0.663
R	78.4 (76/97)	73.1 (49/67)	90 (27/30)	0.069
EMB^*b*^	80.4 (78/97)	74.6 (50/67)	93.3 (28/30)	0.032
INH	9.3 (9/97)	9.0 (6/67)	10.0 (3/30)	1.000
FQ	13.4 (13/97)	17.9 (12/67)	3.3 (1/30)	0.059
DOX	5.2 (5/97)	7.5 (5/7.5)	0 (0/30)	0.320

^*a*^ Data were presented as percentage (numerator/denominator).

^*b*^ DOX, doxycycline; EMB, ethambutol; FQ, fluoroquinolone; INH, isoniazid; M, macrolide; R, rifamycin.

The most commonly used drugs were macrolide (94%, 91/97 patients), ethambutol (80%, 78/97 patients), and rifamycin (78%, 76/97 patients) (Table 2). Ethambutol (*P* = 0.032) was more commonly used in the microbiological cure group than in the non-cure group, but the difference was borderline significant after Bonferroni correction. Among 78 patients treated with ethambutol, 3 (4%) patients had discontinued ethambutol due to adverse effects. Excluding four patients without a recorded body weight, the median daily dose of ethambutol was 15.4 mg/kg (range, 8.9–26.7 mg/kg). Of the 74 patients treated with ethambutol, 44 (59%) received a daily dose ≥15 mg/kg. The microbiological cure group had a longer median treatment duration than the non-microbiological cure group (413.5 days vs 295.5 days, *P* = 0.034). The other characteristics are shown in Table S4, and there were no other significant differences observed between the two groups.

**Fig 1:**
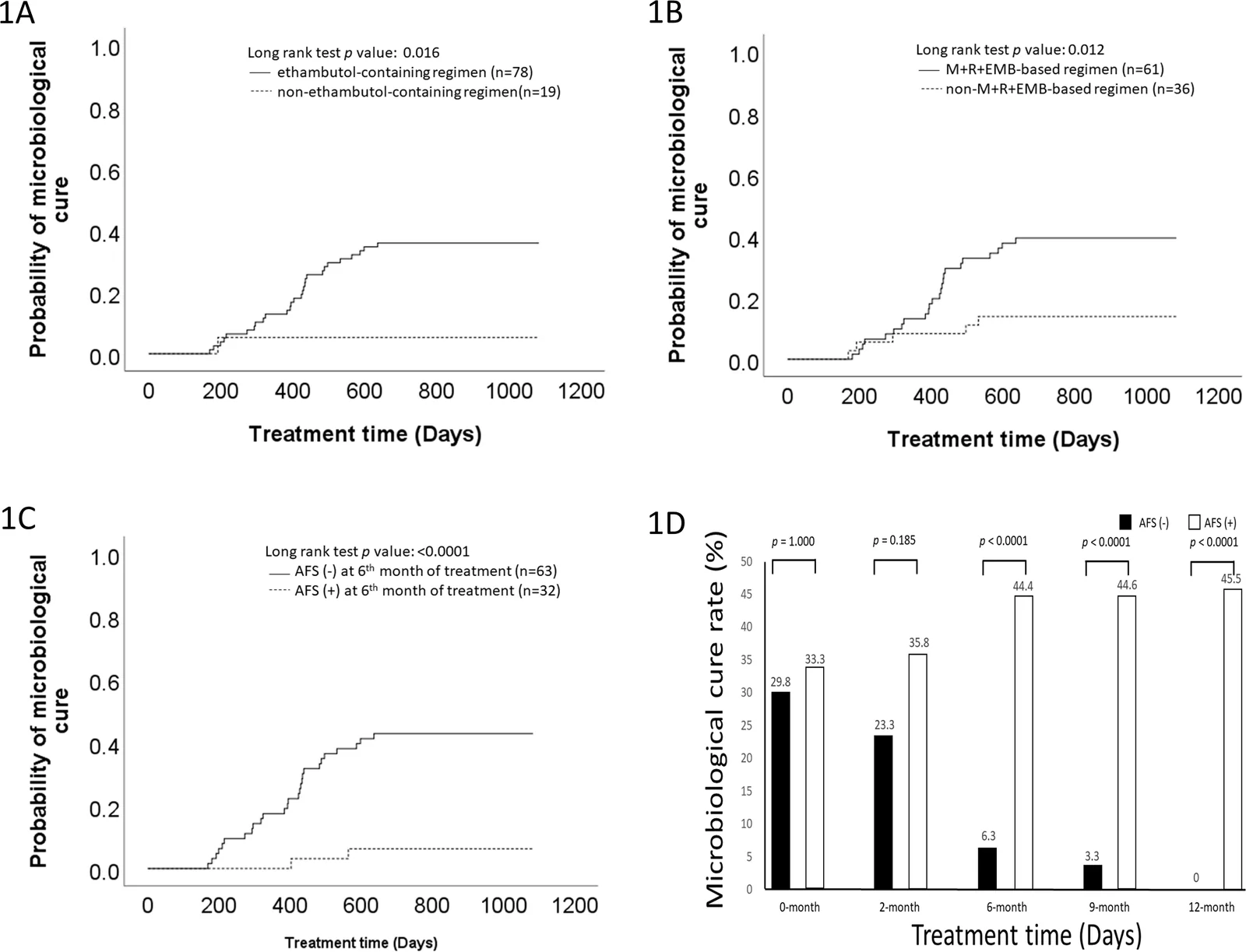
The probability of microbiological cure among patients with *Mycobacterium avium-intracellulare* complex pulmonary disease treated with ethambutol or non-ethambutol (A); macrolide + rifamycin + ethambutol (M + R + EMB)-based or non-M + R + EMB-based regimen (B); AFS(−) or AFS(+) after 6 months of treatment (C) and the microbiological cure rate among the different status of sputum smear (D). AFS, acid fast stain.

Kaplan-Meier analysis further revealed that patients treated with the ethambutol-containing regimen had an increased probability of microbiological cure compared with those treated with the non-ethambutol-containing regimens (log-rank test, *P* = 0.016) (Fig. 1A). Among 91 patients treated with macrolides, 81% (74/91 patients) were also treated with ethambutol. Among MAC-PD patients treated with macrolides, the ethambutol-containing group had a higher microbiological cure rate than the non-ethambutol-containing group (36.5% vs 12%, *P* = 0.049). Among six patients treated without macrolides, 67% (4/6 patients) were also treated with ethambutol. Among MAC-PD patients treated without macrolides, the microbiological cure rate did not differ significantly between ethambutol-containing and non-ethambutol-containing groups (25% vs 0%, *P* = 0.439). The Kaplan-Meier analysis revealed no significant difference in the microbiological cure rate between the group receiving a combination of one or two drugs and the group receiving a combination of three or more drugs (log-rank test, *P* = 0.272) (Fig. S2). Patients treated with the M + R + EMB-based regimen had a higher probability of microbiological cure than those treated with non-M + R + EMB-based regimens (both log-rank tests, *P* = 0.012) (Fig. 1B). There were no differences in the minimum inhibitory concentrations (MICs) between the two groups (Table 3).

**TABLE 3:** Minimum inhibitory concentrations of *Mycobacterium avium-intracellulare* complex isolates to 13 antimicrobial agents

Drug	All (*n* = 97)	Non-microbiological cure (*n* = 67)	Microbiological cure (*n* = 30)	*P* value
	MIC, medium (range)			
CLR^*a*^	2 (0–> 64)	2 (1–64)	2 (0–8)	0.464
AMK	32 (2–64)	32 (2–64)	32 (2–64)	0.636

^*a*^ AMK, amikacin; CLR, clarithromycin; MIC, minimum inhibitory concentration.

There was no significant difference in the microbiological cure rate between patients with initial negative or positive sputum smears (*P* = 0.862). However, the Kaplan-Meier analysis showed that patients with smear-negative sputum after 6 months of treatment had an increased probability of microbiological cure (log-rank test, *P* < 0.001) (Fig. 1C). Patients with persistently positive sputum smears after 6, 9, and 12 months after treatment initiation had significantly lower microbiological cure rates than those with negative sputum smears (all *P* < 0.001; Fig. 1D). Among 32, 30, and 29 patients with persistently positive smears after 6, 9, and 12 months after treatment initiation, 6% (2/32 patients), 3% (1/30 patients), and 0% (0/29 patients) reached culture conversion. None of these patients underwent surgical resection.

We further analyzed the treatment response rate according subspecies, namely, *Mycobacterium avium* subspecies *hominissuis* (MAsH) (25/97, 26%), *Mycobacterium intracellulare* subspecies *intracellulare/chimaera* (MIsI/C) (46/97, 47%), and others (26/97, 27%). The microbiological cure rate was similar between the M + R + EMB-based and non-M + R + EMB-based regimens (log-rank test, *P* = 0.592 for MAsH-PD and 0.093 for MIsI/C-PD) (Fig. S3A and B). Thirty patients with successful treatment were followed up for 1 year. The 1-year relapse rate after treatment completion was 17% (5/30 patients). No significant differences were observed between the relapse and non-relapse groups (Table 4).

**TABLE 4:** Post treatment follow-up in patients with microbiological cure^*a*^

	All (*n* = 30)	Relapse (*n* = 5)	Non-relapse (*n* = 25)	*P* value
Male	43.3 (13/30)	60.0 (3/5)	40 (10/25)	0.628
Age (years)	62.9 (25.8–80.6)	65.1 (41.8–80.6)	62.3 (25.8–78.1)	0.355
BMI^*b*^	18.0 (10.7–25.9) *n* = 26	19.2 (15.6–25.9) *n* = 4	18.0 (10.7–23.9) *n* = 22	0.607
Ex- or current smoker	23.1 (3/13)	50 (1/2)	18.2 (2/11)	
Asthma	0 (0/30)	0 (0/5)	0 (0/25)	
COPD	6.7 (2/30)	20.0 (1/5)	4.0 (1/25)	0.31
Old pulmonary tuberculosis	13.3 (4/30)	40.0 (2/5)	8.0 (2/25)	0.119
Lung cancer	0 (0/30)	0 (0/5)	0 (0/25)	
Hypertension	13.3 (4/30)	20.0 (1/5)	12.0 (3/25)	0.538
Coronary artery disease	10.0 (3/30)	0 (0/5)	12.0 (3/25)	1
Transplantation	3.3 (1/30)	0 (0/5)	4.0 (1/25)	1
Immunosuppressant	0 (0/30)	0 (0/5)	0 (0/25)	
HIV	3.3 (1/30)	0 (0/5)	4.0 (1/25)	1
Type 2 diabetes mellitus	0 (0/30)	0 (0/5)	0 (0/25)	
GERD	3.3 (1/30)	20.0 (1/5)	0 (0/25)	0.167
Rheumatic arthritis	0 (0/30)	0 (0/5)	0 (0/25)	
Pattern				0.713
Fibrocavity	10.0 (3/30)	0 (0/5)	12.0 (3/25)	
Nodular bronchiectasis	73.3 (22/30)	80.0 (4/5)	72.0 (18/25)	
Mixed	16.7 (5/30)	20.0 (1/5)	16.0 (4/25)	
Subspecies				0.693
MAsH	23.3 (7/30)	20 (1/5)	24 (6/25)	
MIsI/C	43.3 (13/30)	60 (3/5)	40 (10/25)	
Others	33.3 (10/30)	20 (1/5)	36 (9/25)	
Time from diagnosis to treatment (days)	105 (4–1,866)	87 (4–798)	109 (13–1,866)	0.448
Positive initial sputum smear	46.7 (14/30)	60.0 (3/5)	44.0 (11/25)	0.642
Time from treatment to sputum smear conversion (days)	80 (7–290)	75 (7–199) *n* = 3	85 (41–290) *n* = 11	0.885
Time from treatment to sputum culture conversion (days)	125.5 (26–422)	199 (26–246)	119 (52–422)	0.914
M + R + EMB-based regimen	80.0 (24/30)	80.0 (4/5)	80.0 (20/25)	1
Treatment duration (d)	399 (169–636)	395 (192–488)	424 (169–636)	0.666
Duration >1 year	63.3 (19/30)	60.0 (3/5)	64.0 (16/25)	1
MIC of major anti-MAC drugs				
CLR	2 (0–8)	2 (1–4)	2 (0–8)	0.786
RMP	8 (1–> 8)	>8 (4–> 8)	8 (1–> 8)	0.447
RFB	0.5 (0.25–8)	1 (0.5–2)	0.5 (0.25–8)	0.447
EMB	8 (4–> 16)	8 (8–16)	8 (4– > 16)	0.377

^*a*^ Data are presented as percentages (numerator/denominator) or medians (range).

^*b*^ BMI, body mass index; CLR, clarithromycin; COPD, chronic obstructive pulmonary disease; HIV, human immunodeficiency virus; GERD, gastroesophageal reflux disease; M + R + EMB, macrolide + rifamycin + ethambutol; MIC, minimum inhibitory concentration; RFB, rifabutin; RMP, rifampin.

## DISCUSSION

In our study, patients with MAC-PD treated with the M + R + EMB-based regimen showed a better microbiological cure rate. Macrolides were the most commonly used drugs for treating MAC-PD. In patients with MAC-PD, sputum smear and culture-negative conversion rates were 40% and 31%, respectively. The overall 3-year microbiological cure rate was 31%. Patients with a negative sputum smear conversion within 6 months had a higher chance of microbiological cure. No differences in microbiological outcomes were found between patients with MAsH and MIsI/C. The 1-year relapse rate after a successful treatment was 17%.

Multidrug combination therapy is potentially effective in the treatment of MAC-PD. Macrolides (azithromycin or clarithromycin), rifamycin, and ethambutol are the most widely accepted guideline-based regimens (8). A regimen that included macrolides was independently and positively associated with sputum conversion and negatively associated with the incidence of refractory disease (15). Macrolide-containing regimens have a better treatment success rate (69%) than those without a macrolides (58.5%) (11). Prevention of macrolide resistance due to inadequate treatment is important (8). Although macrolides are the cornerstone of MAC-PD treatment, no well-designed randomized trials of macrolide treatment in patients with MAC-PD have been performed. The guideline recommends that anti-MAC treatment should be continued for 12 months after conversion to negative conversion (8). Treatment durations of <12 months had similar success rates (66%) to treatment durations of ≥12 months (68%) (11). A South Korean cohort found a 61%–85% sputum culture conversion rate for MAD-PD patients treated with an M + R + EMB-based regimen for ≥12 months (16). However, the evidence supporting this treatment duration is limited. One retrospective study indicated that post-sputum culture conversion treatment for at least 9 months may have similar efficacy as the treatment duration recommended by the guidelines (17). In this study, the median treatment period after sputum culture conversion was only 221.5 days. Only patients with MAC-PD treated with the M + R + EMB-based regimen showed a higher microbiological cure rate. The culture-negative conversion rate in our study was 31%, which was lower than that in previous studies (16). This lower conversion rate may be because only 69% of patients received M + R + EMB-based regimens and the relatively short treatment duration of 324 days.

Macrolides are the cornerstone of anti-MAC regimens (8). Treatment regimens without macrolides were associated with lower sputum culture-negative conversions and higher mortality (9). Azithromycin- and clarithromycin-based regimens have the same efficacy; however, azithromycin-based regimens are recommended over clarithromycin-based regimens owing to fewer drug interactions, single daily dosing, and better intolerance (8). In our study, we demonstrated that azithromycin and clarithromycin were equally effective in MAC-PD treatment. The dose of macrolides (either clarithromycin or azithromycin) might not have an impact on the microbiological cure. In addition to macrolides, ethambutol is the second most important drug because it prevents the development of macrolide resistance. A previous study indicated that the treatment failure rate tended to be higher in patients with MAC-PD who were treated with the standard regimen without ethambutol owing to adverse events and was significantly higher in those who were prescribed fluoroquinolones to replace ethambutol (18). Our study also showed that ethambutol may play a role in a standardized regimen.

The relationship between *in vitro* MAC antibiotic susceptibility and the *in vivo* response to antibiotics is limited. The association between *in vivo* responses and *in vitro* susceptibility to macrolides and amikacin is more evident than *in vitro* susceptibility to rifampin and ethambutol (19). Guidelines recommend susceptibility-based treatment for macrolides and amikacin over empiric therapy (8). However, the poor correlation between treatment outcomes and anti-MAC drugs, except for macrolides and amikacin, may be due to limited evidence. In our study, there were no differences in the anti-MAC MICs between the microbiologically cured and non-cured groups.

The outcomes of macrolide-containing regimens in patients with MAC-LD were unsatisfactory. Low BMI, human immunodeficiency virus, positive smear at the start of treatment, presence of a cavity, and non-guideline-based treatment have been reported as poor prognostic factors for the management of MAC-PD (11, 20). *Mycobacterium avium* and *Mycobacterium intracellulare* are the two major subspecies, with the latter being more virulent (3) and may have different treatment outcomes. However, available data are limited. In our study, no differences in the microbiological outcomes were found between the MAsH and MIsI/C treatments. However, the population size was small, and further investigation is required.

Evidence has shown a high relapse rate despite MAC-PD therapy for ≥24 months (21). The high relapse rate may be due to exposure to environmental sources such as water or soil, which facilitate reinfection (22). The MAC-PD recurrence rate after successful treatment ranges from 8.3% to 48% (23). Discrepancies in recurrence rates may be due to different anti-MAC regimens, treatment durations, disease severity, and follow-up periods after completion of therapy. Many risk factors for recurrence have been identified, such as longer intervals between diagnosis and treatment initiation, increased number of involved lobes, nodular bronchiectatic pattern, failure of sputum culture conversion within 6 months, and receiving less than 15 months of treatment following culture conversion (23). In our study, the 1-year relapse rate was 17%. No risk factors for relapse were identified due to the small sample size. Therefore, further studies with larger sample sizes are needed.

Our study had several limitations. First, it had a small sample size and included patients from a single center. Second, the frequency of drug administration was not evaluated in the present study. Third, the clinical response to anti-MAC treatment was not evaluated. Fourth, treatment initiation and regimens were assessed by the attending pulmonary physicians. Physician bias could potentially influence prognosis. Fifth, since this was a retrospective study, repeated sputum smears/cultures for MAC were judged by clinical physicians, and no standard protocol was used. Sixth, we did not check the concentration of macrolides; therefore, we were not sure whether macrolide concentration is the key pharmacodynamic parameter in MAC-PD treatment. Seventh, we did not check the MIC of the isolate in the non-microbiological conversion cases. Thus, we could not determine whether the non-microbiological conversion was caused by acquired macrolide resistance. Eighth, reinfection was defined as infection with a different strain of the causative species, and recurrence was defined as infection with the same strain of the species. Because identification of the relapsed species was not compared with that of previously infected species, we could not discriminate between reinfection and recurrence. Ninth, the prescriptions in this study were determined by clinicians based on the treatment guidelines and patient conditions. However, this study did not investigate how clinicians made these decisions.

In conclusion, the M + R + EMB-based regimen and sputum smear-negative conversion within 6 months were associated with a higher rate of microbiological cure. Patients with persistently positive sputum smears after 6 months of treatment had a lower microbiological cure rate. The 1-year relapse rate after a successful treatment was 17%.

## MATERIALS AND METHODS

### Setting, participants, and data collection

This retrospective study was conducted at the National Taiwan University Hospital, a tertiary care center in northern Taiwan. All consecutive patients diagnosed with MAC-PD between April 2011 and December 2020 according to the 2020 American Thoracic Society/Infectious Diseases Society of America guidelines (24) were enrolled. The diagnostic criteria included clinical and microbiological sections as previously described (3). All antibiotic-naïve participants were treated with anti-MAC medication, judged by clinical physicians according to the severity of MAC-PD, risk of progressive MAC-PD, and presence of comorbidities (5), and were followed up for 3 years after treatment initiation. In this retrospective analysis, the treatment regimen was modified by a primary physician, and the patients were grouped according to the first regimen of MAC-PD treatment. At our hospital, we use the Ishihara test to evaluate and monitor optic neuropathy during clinical visits in all patients treated with ethambutol. Patients with a microbiological cure were followed up for 1 year after treatment completion and assessed for relapse after 1 year.

### Smear microscopy, culture, and identification of MAC species and subspecies

Smear microscopy and *Mycobacterium* culture were performed as previously described (25). Each respiratory specimen was processed by adding an equal volume of NaOH-citrate-N-acetyl-l-cysteine at room temperature for 15 minutes. After centrifugation, the precipitate was resuspended in 1 mL of phosphate-buffered saline. Smears were analyzed using the Ziehl-Nielsen staining. All isolates were cultured in BACTEC MGIT tubes (Becton-Dickinson, Sparks, USA) and Lowenstein-Jensen medium slant (Becton-Dickinson) before further testing. MAC isolates were identified using conventional biochemical methods.

### Radiographic severity and outcome evaluation

Chest radiographs and high-resolution computed tomography scans were reviewed by a trained chest specialist and thoracic radiologist who were blinded to the clinical data. If their interpretations differed, the images were further evaluated by a third blinded pulmonologist. The disease extent assessed on initial and follow-up images was interpreted as the radiographic score (26). Briefly, each lung was divided into three areas, and each area was rated on a four-point scale of 0 to 3 for the extent of infiltration, including any lung lesion, such as a cavity, with a maximum radiographic score of 18. Higher scores indicate greater disease involvement. Radiographic progression was defined as an increase in the radiographic score by at least one point. The image pattern was classified as fibrocavity, nodular bronchiectasis, or mixed type.

Negative smear/culture conversion was defined as three consecutive negative smears/cultures over a period of at least 2 months (27). After achieving culture conversion with three consecutive negative cultures, a microbiological cure requires three consecutive negative cultures until treatment completion (28). Relapse was defined as the occurrence of two or more positive cultures with the same strain of causative species after the cessation of treatment, regardless of the radiographic appearance (28).

### Antimicrobial susceptibility testing

The first MAC isolate obtained from the patient with NTM-PD was used for antimicrobial susceptibility testing. The MICs of the isolates were determined using the SLOMYCO Sensititre panel (TREK Diagnostic Systems, Cleveland, OH, USA) (29), and the culture medium was Middlebrook 7H9.

### Statistical analysis

Statistical analyses were performed using SPSS version 25.0 (IBM Corp., Armonk, NY, USA). Continuous variables were reported as medians and ranges. Between-group differences in continuous variables were compared using the Mann-Whitney *U* test. Categorical variables were expressed as counts and percentages. Differences between categorical variables were compared using the chi-square test. Bonferroni correction for multiple comparisons was used for calculating confidence intervals (99.2% confidence interval for a total of six drug efficacy outcome classifications). Microbiological outcomes between different groups were estimated using Kaplan-Meier analysis and compared using the log-rank test. Statistical tests were two sided, and significance was set at *P* <0.05.

## SUPPLEMENTAL MATERIAL

Supplemental material spectrum.01640-23-s0001.docx Tables S1 to S4 and Fig. S1 to S3.

ASM does not own the copyrights to Supplemental Material that may be linked to, or accessed through, an article. The authors have granted ASM a non-exclusive, world-wide license to publish the Supplemental Material files. Please contact the corresponding author directly for reuse.
