# Supplementary material for: Treatment outcomes and relapse in patients with Mycobacterium avium-intracellulare complex pulmonary disease
Source: Microbiol Spectr. 2023 Sep 27;11(5):e01640-23. doi: 10.1128/spectrum.01640-23 (PMC10581154; doi:10.1128/spectrum.01640-23)
Supplement: Supplemental material — Tables S1 to S4 and Fig. S1 to S3. [file spectrum.01640-23-s0001.docx]

**Supplementary table 1. Treatment regimens in patients with *Mycobacterium avium-intracellulare complex* pulmonary disease**

| **Treatment Regimen** | **All**  **(n=97)** | **Non- microbiological cure**  **(n=67)** | **Microbiological cure**  **(n=30)** | ***p* value** |
| --- | --- | --- | --- | --- |
| M+R+EMB-base | 62.9 (61/97) | 55.2 (37/67) | 80.0 (24/30) | 0.024 |
| M+R+EMB | 55.7 (54/97) | 47.8 (32/67) | 73.3 (22/30) | 0.027 |
| M+R+EMB+FQ | 4.1 (4/97) | 6.0 (4/67) | 0 (0/30) | 0.308 |
| M+R+EMB+DOX | 1.0 (1/97) | 1.5 (1/67) | 0 (0/30) | 1.000 |
| M+R+EMB+INH | 2.1 (2/97) | 0 | 6.7 (2/30) | 0.093 |
| M+EMB+INH+FQ | 1.0 (1/97) | 1.5 (1/67) | 0 (0/30) | 1.000 |
| M+EMB+FQ | 2.1 (2/97) | 1.5 (1/67) | 3.3 (1/30) | 0.525 |
| M+EMB+INH | 3.1 (3/97) | 4.5 (3/67) | 0 (0/30) | 0.550 |
| M+EMB | 7.2 (7/97) | 7.5 (5/67) | 6.7 (2/30) | 1.000 |
| M+R+FQ | 6.2 (6/97) | 7.5 (5/67) | 3.3 (1/30) | 0.663 |
| M+R+INH | 1.0 (1/97) | 1.5 (1/67) | 0 (0/30) | 1.000 |
| M+R | 4.1 (4/97) | 4.5 (3/67) | 3.3 (1/30) | 1.000 |
| M+FQ+DOX | 3.1 (3/97) | 4.5 (3/67) | 0 (0/30) | 0.550 |
| M+FQ | 2.1 (2/97) | 3.0 (2/67) | 0 (0/30) | 1.000 |
| M | 1.0 (1/97) | 1.5 (1/67) | 0 (0/30) | 1.000 |
| R+EMB+INH | 2.1 (2/97) | 1.5 (1/67) | 3.3 (1/30) | 1.000 |
| R+EMB+FQ | 1.0 (1/97) | 1.5 (1/67) | 0 (0/30) | 1.000 |
| R+EMB | 1.0 (1/97) | 1.5 (1/67) | 0 (0/30) | 1.000 |
| FQ | 1.0 (1/97) | 1.5 (1/67) | 0 (0/30) | 1.000 |
| FQ+DOX | 1.0 (1/97) | 1.5 (1/67) | 0 (0/30) | 1.000 |
| Time from diagnosis to treatment (days) | 79.0 (0-1866) | 56 (0-1715) | 105 (4-1866) | 0.288 |
| Treatment duration (days) | 324 (30-1095) | 273 (30-1095) | 413.5 (169-1044) | 0.016 |
| <0.5 year | 23.7 (23/97) | 31.3 (21/67) | 6.7 (2/30) | 0.009 |
| <1 year | 55.7 (54/97) | 64.2 (43/67) | 36.7 (11/30) | 0.015 |
| <2 year | 93.8 (91/97) | 94.0 (63/67) | 93.3 (28/30) | 1.000 |

Data were presented as percentage (numerator/denominator)

Abbreviations: DOX, doxycycline; EMB, ethambutol; FQ, fluoroquinolone; INH, isoniazid; M, macrolide; R, rifamycin.

**Supplementary table 2. Treatment responses of patients with *Mycobacterium avium-intracellulare complex* pulmonary disease.**

|  | **All**  **(n=97)** | **Non- microbiological cure**  **(n=67)** | **Microbiological cure**  **(n=30)** | ***p* value** |
| --- | --- | --- | --- | --- |
| Positive initial sputum smear | 49.5 (47/95) | 50.8 (33/65) | 46.7 (14/30) | 0.862 |
| Sputum smear conversion | 40.4 (19/47) | 15.2 (5/33) | 100 (14/14) | <0.0001 |
| 1-month sputum smear conversion | 2.1 (1/47) | 0 (0/33) | 7.1 (1/14) | 0.298 |
| 2-month sputum smear conversion | 8.5 (4/47) | 0 (0/33) | 28.6 (4/14) | 0.006 |
| 6-month sputum smear conversion | 31.9 (15/47) | 9.1 (3/33) | 85.7 (12/14) | <0.0001 |
| Time from treatment to sputum smear conversion (days) | 111 (7-438) | 136 (84-438) | 80 (7-290) | 0.156 |
| Sputum culture conversion | 30.9 (30/97) | 0 | 100 (30/30) | - |
| 2-month sputum culture conversion | 4.1 (4/97) | - | 13.3 (4/30) | - |
| 6-month sputum culture conversion | 21.6 (21/97) |  | 70 (21/30) | - |
| 12-month sputum culture conversion | 28.9 (28/97) |  | 93.3 (28/30) | - |
| Time from treatment to sputum culture conversion, days | 125.5 (26-422) | - | 125.5 (26-422) | - |
| Time from sputum culture conversion to complete treatment, days | 221.5 (0-483) | - | 221.5 (0-483) | - |
| Duration >1 years | 16.7 (5/30) | - | 16.7 (5/30) | - |

Data are presented as percentages (numerator/denominator) or medians (range).

**Supplementary table 3. Medications for patients not prescribed with macrolides.**

| **Treatment Regimen** | **Patient number** |
| --- | --- |
| rifamycin + ethambutol + isoniazid | 2 |
| rifamycin+ethambutol+ fluoroquinolone | 1 |
| rifamycin + ethambutol | 1 |
| fluoroquinolone | 1 |
| fluoroquinolone + doxycycline | 1 |

**Supplementary table 4. The characteristics of patients treated with ethambutol**

|  | Non-microbiological cure (n=50) | Microbiological cure (n=28) | P value |
| --- | --- | --- | --- |
| Male | 46 (23/50) | 42.9 (12/28) | 0.817 |
| Age, years | 59.9 (24.4-87.4) | 61.9 (25.8-78.1) | 0.950 |
| BMI | 19.1 (11.8-26.9) n=47 | 17.8 (10.7-29.3) n=24 | 0.226 |
| Image  Fibro-cavity  Nodular bronchiectasis  Mixed type | 18 (9/50)  68 (34/50)  14 (7/50) | 10.7 (3/28)  71.4 (20/28)  17.9 (5/28) | 0.663 |
| Subspecies  MAsH  MIsI/C  Others | 30 (15/50)  48 (24/50)  22 (1/50) | 25 (7/28)  42.9 (12/28)  32.1 (9/28) | 0.612 |
| M+R+EMB-base | 74 (13/50) | 85.7 (24/28) | 0.267 |
| Time from treatment to sputum culture conversion, days | - | 132 (52-422) | - |
| Treatment time (days) | 295.5 (30-1095) | 413.5 (169-1044) | 0.034 |
| Treatment duration > 1 year | 36 (18/50) | 64.3 (18/28) | 0.020 |

**Supplementary figure 1. The probability of culture conversion and microbiological cure among patients treated with different dose of clarithromycin (Panel 1A and 1B) and azithromycin (Panel 1C and 1D).**


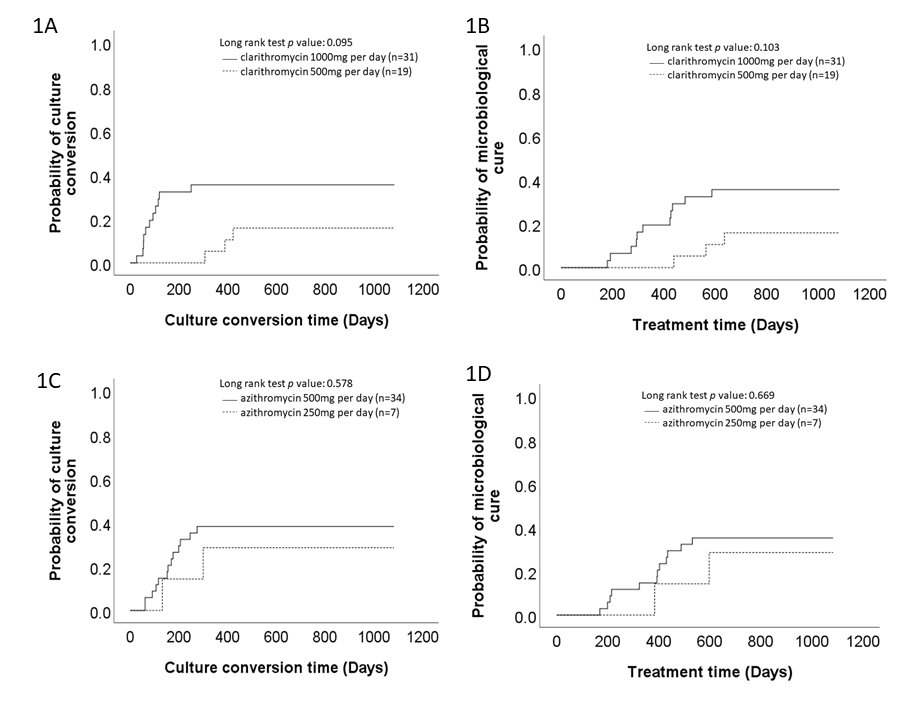


**Supplementary figure 2. The probability of microbiological cure among patients treated with the group receiving a combination of one or two drugs and the group receiving a combination of three or more drugs.**

**
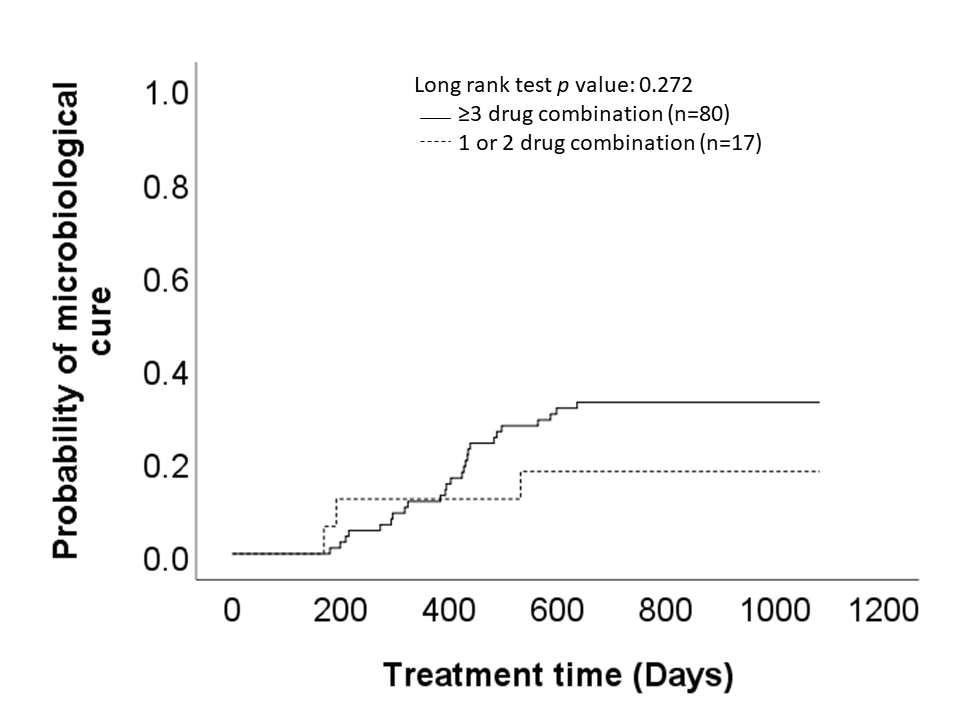
**

**Supplementary figure 3. The probability of microbiological cure among patients with MAsH (Panel A) and MIsI/C-PD (Panel B), treated with M+R+EMB-base and non-M+R+EMB base regimen.** EMB, ethambutol; M, macrolide; MAsH, *mycobacterium avium* subspecies *hominissuis*; MIsI/C, *mycobacterium intracellulare* subspecies *intracellulare/chimaera*; PD, pulmonary disease; R, rifamycin.

**
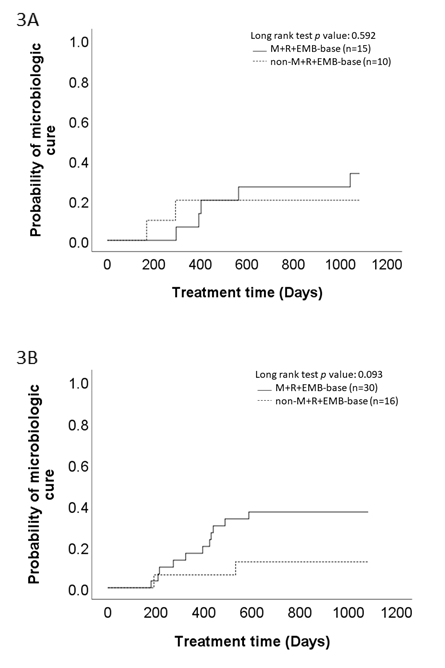
**
